# The Metabolism of *Clostridium ljungdahlii* in Phosphotransacetylase Negative Strains and Development of an Ethanologenic Strain

**DOI:** 10.3389/fbioe.2020.560726

**Published:** 2020-10-27

**Authors:** Jonathan Lo, Jonathan R. Humphreys, Joshua Jack, Chris Urban, Lauren Magnusson, Wei Xiong, Yang Gu, Zhiyong Jason Ren, Pin-Ching Maness

**Affiliations:** ^1^National Renewable Energy Laboratory, Golden, CO, United States; ^2^Andlinger Center for Energy and Environment, Princeton University, Princeton, NJ, United States; ^3^Key Laboratory of Synthetic Biology, CAS Center for Excellence in Molecular Plant Sciences, Shanghai Institute of Plant Physiology and Ecology, Chinese Academy of Sciences, Shanghai, China

**Keywords:** syngas, acetogen, metabolic engineering, CO_2_ fixation, *Clostridium ljungdahlii*, autotrophic

## Abstract

The sustainable production of chemicals from non-petrochemical sources is one of the greatest challenges of our time. CO_2_ release from industrial activity is not environmentally friendly yet provides an inexpensive feedstock for chemical production. One means of addressing this problem is using acetogenic bacteria to produce chemicals from CO_2_, waste streams, or renewable resources. Acetogens are attractive hosts for chemical production for many reasons: they can utilize a variety of feedstocks that are renewable or currently waste streams, can capture waste carbon sources and covert them to products, and can produce a variety of chemicals with greater carbon efficiency over traditional fermentation technologies. Here we investigated the metabolism of *Clostridium ljungdahlii*, a model acetogen, to probe carbon and electron partitioning and understand what mechanisms drive product formation in this organism. We utilized CRISPR/Cas9 and an inducible riboswitch to target enzymes involved in fermentation product formation. We focused on the genes encoding phosphotransacetylase (*pta*), aldehyde ferredoxin oxidoreductases (*aor1* and *aor2*), and bifunctional alcohol/aldehyde dehydrogenases (*adhE1* and *adhE2*) and performed growth studies under a variety of conditions to probe the role of those enzymes in the metabolism. Finally, we demonstrated a switch from acetogenic to ethanologenic metabolism by these manipulations, providing an engineered bacterium with greater application potential in biorefinery industry.

## Introduction

Acetogenic bacteria utilize the Wood-Ljungdahl pathway (WLP) to non-photosynthetically fix inorganic carbon into acetyl-CoA, which is then converted into products, normally acetate and to a lesser extent, ethanol. Acetate production is an important feature of acetogenic metabolism. In *Clostridium ljungdahlii*, a model acetogen, acetate formation primarily occurs sequentially with the conversion of acetyl-CoA to acetyl-P via phosphotransacetylase (PTA) and then from acetyl-P to acetate via acetate kinase (ACK), with the ACK step generating ATP (Köpke et al., 2010). Acetate generation is important for ATP formation, as acetogens have low ATP budgets because they survive on the “thermodynamic limit of life”, on a small free energy change (Schuchmann and Müller, 2014).

Based on genome information and published data, *C. ljungdahlii* is believed to make ethanol from acetyl-CoA via two established mechanisms: aldehyde ferredoxin oxidoreductase (AOR) and bifunctional aldehyde/alcohol dehydrogenase (AdhE) (Köpke et al., 2010; Leang et al., 2013). AOR catalyzes the reversible conversion of acetate to acetaldehyde, with ferredoxin as the electron acceptor/donor. AdhE is a bifunctional enzyme, catalyzing the reversible conversion of acetyl-CoA to acetaldehyde, then to ethanol, using NAD(P)H as the electron donors.

Acetogens have been studied for their ability to generate value-added products from either syngas alone or in conjunction with sugar for increased carbon yield (Köpke et al., 2010; Jones et al., 2016). Many of these value-added products are derived from acetyl-CoA, which is generated from the WLP and glycolysis and serves as a primary intermediate in central carbon metabolism. Thus, it would be useful to understand the metabolism around this acetyl-CoA node to help guide metabolic engineering efforts.

Pta is the most obvious target, as it is the first and direct step to making acetate from acetyl-CoA. *C. ljungdahlii pta* (CLJU_c12770) has been targeted in three studies via gene disruption, CRISPR interference, and CRISPR/Cas9, but in all cases it was ambiguous what was happening. In the gene disruption study of *pta*, *pta* was replaced with a butyrate pathway that could have side reactions to generate acetate (Ueki et al., 2014). In the CRISPR interference study, knockdown of *pta* was incomplete and did not show an obvious growth phenotype leaving open the possibility that leaky expression was responsible for acetate production (Woolston et al., 2018). In the CRISPR/Cas9 study, the *pta* deletion strain growth data was only characterized vs. the wild type on syngas, showing no significant growth after 96 h or characterization of heterotrophic growth (Huang et al., 2016). High yields of acetate remained in strains targeting *pta*, suggesting that alternative pathways may be important for acetate production. Studies of acetyl-CoA metabolism suggest that there may be multiple important enzymes and pathways utilizing acetyl-CoA (Ueki et al., 2014; Whitham et al., 2015; Huang et al., 2016). Thus, we set out to delete *pta* and other key genes to understand their roles in metabolism and product formation.

## Materials and Methods

### Microbial Strains and Media Composition

*Clostridium ljungdahlii* DSM 13528 was acquired from The Leibniz Institute DSMZ (Braunschweig, Germany). Routine growth was performed in YTF media (10 g L^–1^ yeast extract, 16 g L^–1^ Bacto tryptone, 4 g L^–1^ sodium chloride, 5 g L^–1^ fructose, 0.5 g L^–1^ cysteine, pH 6) at 37°C with a N_2_ headspace. Routine manipulations and growth were performed in a COY (Grass Lake, MI) anaerobic growth chamber, flushed with 95% N_2_ and 5% H_2_ and maintained anaerobic by palladium catalyst.

For growth and fermentation characterization, cells were grown anaerobically in PETC 1754 media (ATCC) with sodium sulfide omitted. The gas pressure was at 1 atm, and the headspace was flushed with N_2_ unless indicated otherwise. Cells were grown in 18 × 150 mm Balch tubes (approximate total volume of 29 mL) crimped shut with 5 mL of PETC, with fructose, CO, or H_2_ added as described, standing upright in a 37°C incubator, shaking at 250 RPM. All fermentation data shown was the result of three replicates. Media components were supplied by Sigma-Aldrich (St. Louis, MO), while gases were > 99% purity, supplied by Airgas (Radnor Township, PA).

*Escherichia coli* strains were acquired from New England Biolabs (NEB) (Ipswich, MA). NEB 10-beta was utilized for general molecular purposes and plasmid propagation, while *E. coli* strain NEB Express was used for plasmid preparation for transformation into *C. ljungdahlii* due to methylation compatibility (Leang et al., 2013).

### Molecular Techniques

Standard molecular techniques were used with enzymes from NEB. For routine PCR and cloning, Phusion polymerase was used to create and amplify fragments. For PCR screening the genetic loci of target genes, LongAmp polymerase was used. The ladder used was 1 kb Opti-DNA Marker from Applied Biological Materials (Vancouver, Canada). To generate new constructs, DNA was ligated together using Gibson assembly (NEB).

Plasmids from the pMTL80000 modular system from Chain Biotech (Nottingham, United Kingdom) were used to generate the constructs transformed into *C. ljungdahlii*. The original construct with Cas9 targeting *pta* was acquired from the original paper’s author describing the Cas9 system in *C. ljungdahlii* and ligated into pMTL83151 to generate the *pta* deletion construct (Huang et al., 2016). The plasmids were retargeting accordingly by ligating the new guide RNA and homology arms via PCR overlap extension (plasmids and primers are listed in Supplementary Table S1).

Complement plasmid for the *aor2* and *pta/ack* were created using HiFi assembly (NEB) as follows. Gene fragments were gel purified from PCR amplification using Phusion DNA polymerase and the primers stated in Supplementary Table S1. pMTL83151 was cut using *Not*I and gel purified. Purified gene fragments and *Not*I cut pMTL83151 were assembled using HiFi assembly following the manufacturer’s instructions. Resulting colonies were screened with the primers used to make the gene fragments. Positive colonies were grown overnight, and plasmids purified using Monarch plasmid miniprep kit (NEB). Confirmation of complete plasmids was confirmed by whole plasmid sequencing by MBH DNA Core (Cambridge, MA).

### Electrocompetent Cell Preparation and Transformation Protocol

Transformation protocols were similar to previously reported conditions (Leang et al., 2013). Briefly, *C. ljungdahlii* was grown on YTF with 40 mM DL-Threonine to early log phase (0.2–0.7), harvested and washed with ice cold SMP buffer (270 mM sucrose, 1 mM MgCl_2_, 7 mM sodium phosphate, pH 6), then resuspended in SMP buffer with 10% (dimethyl sulfoxide) DMSO and frozen at −80°C until use for transformation.

Electroporation was performed in a COY chamber with a Bio-Rad (Hercules, California) Gene Pulser Xcell electroporator under the following conditions: 25 μL cells were mixed with 2–10 μg of DNA in a 1 mm cuvette, pulsed 625 kV with resistance at 600 Ω and a capacitance of 25 μF. They were then resuspended in 5 mL of YTF and recovered overnight at 37°C. Cells were then plated in 1.5% agar YTF with thiamphenicol (Tm) at a final concentration of 10 μg/mL, which was Tm concentration used for general propagation. Typically, colonies appeared between 3 and 7 days after plating.

### Generating and Validating Deletion Strains

To generate the plasmids targeting *pta* (CLJU_c12770) NC_014328.1 (1376316.1377317), we received the *pta* targeting Cas9 fragment described from Huang et al. (2016) and recreated the *pta* deletion vector by cloning in the fragment via restriction cloning into pMTL83151. Subsequent *aor2* (CLJU_c20210) (2192715.2194538), and *adhE1*/*adhE2* (CLJU_c16510/CLJU_c16520) (1791269.1793881/1794033.1796666), Cas9 deletion constructs were generated with the *pta* targeting vector as a backbone, using PCR to change gene targeting, similar to previously reported protocols (Huang et al., 2016). For the *aor1* (CLJU_c20110) (2179645.2181468) deletion, the original promoter driving *cas9* was replaced with a riboswitch-linked promoter (pbuE, *P* = 8 bp) activated by 2-aminopurine (2-AP) at 2 mM (Marcano-Velazquez et al., 2019). These strains were dilution plated on YTF agar with Tm and 2-AP and allowed for grow until colonies appeared (around 1 week). Once colonies appeared, they were individually picked, and PCR screened to isolate bacteria with successful genetic editing. To cure the plasmid and restore sensitivity to Tm, these strains were serially transferred 2 times in non-selective media and dilution plated on YTF agar for single colony isolation, then tested for sensitivity to Tm and PCR screened for loss of the Tm resistance gene.

### Analytical Techniques

Fermentation liquid samples of 150 μL were extracted by syringe, filtered using Costar Spin-X 0.45 μm filters (Corning, Corning, NY), and stored at −20°C until the experiments completed. Fermentation products in the liquid phase (acetate, ethanol, and 2,3 butanediol) were measured by high-performance liquid chromatography on a 1,200 series Agilent (Santa Clara, CA) with an Aminex HPX-87H column using a Micro Guard Cation H Cartridge, at 55°C with 4 mM H_2_SO_4_ mobile phase, as previously described (Marcano-Velazquez et al., 2019). Optical density was measured at 600 nm using a Milton Roy (Ivyland, PA) Spectronic 21D.

### Biochemical Assays

Wild type and derived cultures were grown in 50 mL PETC medium with 5 g/L fructose and harvested at mid log phase (0.3–0.7 OD_600_) by centrifugation at 4,000 g for 10 min. The supernatants were discarded, and pellets were frozen at −80°C for future use. Cell pellets were resuspended in 200 μL phosphate buffer (pH 7.6) which contained 25 mg/ml lysozyme and 10 mM dithiothreitol. Resuspended cells were added to a screwcap tube contained acid washed glass beads (Sigma-Aldrich, United States) and lysed using a Tissuelyser II (Qiagen, Germany) at a frequency of 30 1/s for 2 min. Lysed cells were subsequently centrifuged at 17,000 g for 2 min. The resulting supernatant was extracted and stored in anaerobic vials at –80°C for future use. Protein concentration was determined using Bradford reagent (Sigma-Aldrich) following the manufacturer’s instructions using a Tecan infinite M200 pro plate reader (Tecan Life Sciences, Switzerland). All assays are reported as specific activities (μmol min^–1^ mg^–1^) and were performed in triplicate in the anaerobic COY chamber at 27°C. Enzyme assay mixtures without cell free extract were used to track baseline changes in absorbance.

To test Pta activity in the wild type and derived mutants, the procedure employed was based on formation of thioester bond formation of acetyl-CoA, following the change in absorbance at 233 nm using a molar extinction coefficient of 4,360 M^–1^ cm^–1^ (Klotzsch, 1969). In short, 200 μL of a 100 mM Tris-HCl (pH 7.6) containing 1.6 mM glutathione, 0.43 mM coenzyme-A (CoA), 7.23 mM acetyl-phosphate, and 13.3 mM ammonium sulfate was added to 1 μL of cell free extract, and monitored for 233 nm change in a NanoDrop One from Thermo Fisher Scientific (Waltham, MA) at 15 s intervals for 4 min.

To test Aor activity in the wild type and derived mutants, the procedure employed was based on benzyl viologen reduction (Heider et al., 1995). In short, 200 μL of a 100 mM phosphate buffered mix (pH 7.6) containing 1.6 mM benzyl viologen, 1 mM acetaldehyde and 1 mM dithiothreitol was added 10 μL of cell free extract in a flat bottom 96 well plate. Reduction of benzyl viologen was measured at 600 nm using a molar extinction coefficient of 7,400 M^–1^ cm^–1^ using BioTek Synergy Neo2 plate reader (BioTek Instruments, United States) at 10 s intervals for 5 min.

To test both Adh and Aldh activity in the wild type and derived mutants, enzymatic reduction of NAD^+^/NADP^+^ was employed based on previous published methods (Lo et al., 2015). For the Adh assay, 200 μL of a 100 mM Tris-HCL (pH 7.8) buffer mixture containing 10 mM ethanol, 1 mM NAD^+^ or 1 mM NADP^+^ and 1 mM dithiothreitol was added to 10 μL of cell free extract in a flat bottom 96 well plate. Reduction of NAD^+^ or NADP^+^ was measured at 340 nm using BioTek Synergy Neo2 plate reader at 10 s intervals for 5 min. For the Aldh assay, 200 μL of 100 mM Tris-HCL (pH 7.8) buffer mixture containing 0.43 mM CoA, 1 mM NAD^+^ or 1 mM NADP^+^, 10 mM acetaldehyde and 1 mM dithiothreitol was added to 10 μL of cell free extract in a flat bottom 96 well plate. Reduction NAD(P)^+^ was calculated using a molar extinction coefficient of 6,220 M^–1^ cm^–1^.

## Results

### Generation and Characterization of Phosphotransacetylase Gene(*pta*) Negative Strain

**FIGURE 1:**
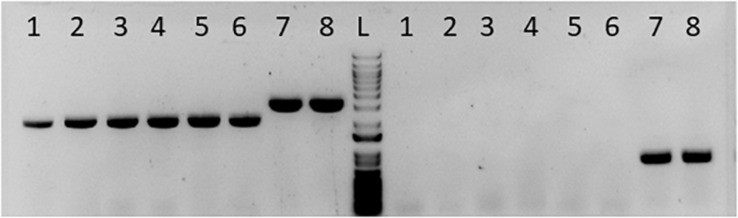
Gel picture showing successful deletion of *pta* in *C. ljungdahlii*. Columns labeled 1–6 to the left of the ladder “L” were presumptive *pta* deletion colonies for single colony purification. Column 7 was wild type DNA mixed with the *pta* deletion plasmid. Column 8 is the wild type DNA alone. The set of lanes to the left of the ladder “L” was PCR amplifying the *pta* region. Columns 1–6 show a smaller *pta* region, indicating loss of *pta* at that region in the genome. Columns 7 and 8 show the wild type band size. The set of lanes to the right of the ladder (L) was PCR amplifying a region inside *pta*. Columns 1–6 show no fragment amplified, indicating no detectable *pta* in those colonies. Columns 7 and 8 show the fragment of *pta* in unedited DNA.

To determine the role of *pta* in acetate formation, we utilized Cas9 to delete *pta*. Cas9 can be utilized to generate markerless deletion strains, which is useful for generating strains with multiple deletions when selective markers are limited. Because of the importance of *pta* to acetogenic metabolism and as a target for genetic engineering for increased product formation, we acquired the Cas9 *pta* deletion cassette and with which created the *pta* deletion strain (Figure 1).

**FIGURE 2:**
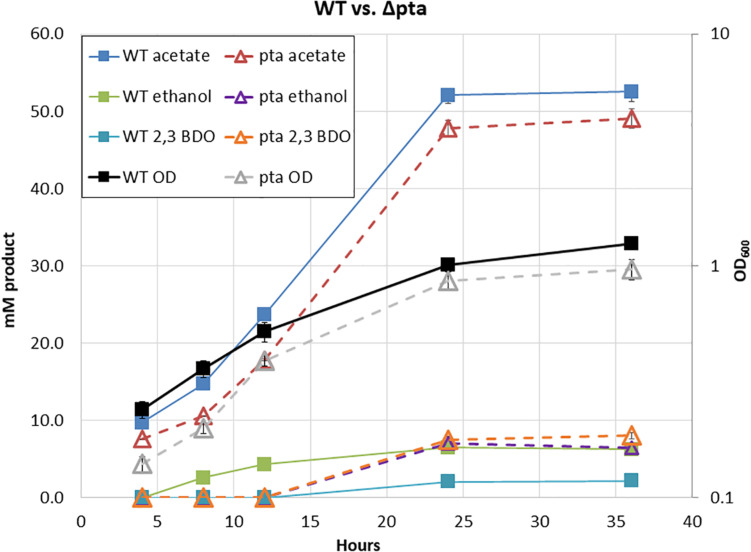
Heterotrophic growth and fermentation products of *C. ljungdahlii* wild type and the *pta* deletion strain on PETC with 5 g/L fructose. Wild type is marked with solid lines and squares, while Δ*pta* is marked dashed lines with hollow triangles. Growth rate, OD_600_, and fermentation products are similar across the duration of the experiment.

Surprisingly, in our initial characterization of heterotrophic growth, there was little initial difference between the Δ*pta* and wild-type strain (Figure 2). The wild type and Δ*pta* strain grew at similar rates and produced a similar amount of products. Both the wild type and Δ*pta* strain produced high levels of acetate during heterotrophic growth (51 vs. 47 mM) and similar amounts of ethanol (∼6 mM). The only notable difference is that the Δ*pta* strain produced markedly more 2,3 butanediol (2,3 BDO) than the wild-type strain (8 vs. 2 mM). The carbon/electron recovery from fermentation products was 0.79/0.85 for the wild type and 0.86/0.97 for the Δ*pta* strain.

Predictions from the metabolic model developed by Nagarajan et al. (2013) suggested loss of *pta* would redirect flux toward ethanol formation through AdhE, yet we observed no increase in ethanol. We reasoned that if acetyl-CoA was no longer being directed toward acetate via acetyl-P, perhaps it was through an acetaldehyde intermediate via Aldh and AOR. If this was the case, NADH would be consumed, ferredoxin would be reduced, and acetaldehyde would be oxidized to acetate. The increased reduced ferredoxin (Fd_red_) could then be utilized by the WLP to fix the CO_2_ evolved by glycolysis. Redox consumption via the WLP may be preventing ethanol formation.

### Creation and Characterization of Δ*pta* Derivative Strains

**FIGURE 3:**
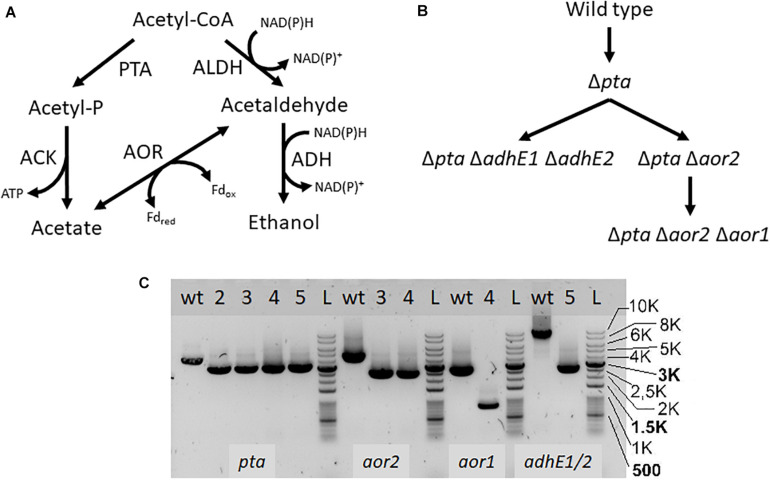
Acetyl-CoA metabolism and creation of deletion mutants. PTA, phosphotransacetylase; ACK, acetate kinase; ALDH/ADH, aldehyde/alcohol dehydrogenase; AOR, aldehyde ferredoxin oxidoreductase **(A)** Map of acetyl-CoA metabolism with metabolites, cofactors, and enzyme reactions. **(B)** Strain map showing lineage of deletions made. **(C)** PCR confirmation of deletion strains. Primers were used to amplify the genomic regions of *pta*, *aor2*, *aor1*, *adhE1*, and *adhE2*, to confirm genomic deletions. Lanes labeled with “wt” is wild type DNA control, while lanes with “L” are the DNA ladder. Lanes with numbers correspond to the following strains: 2- Δ*pta*, 3- Δ*pta* Δ*aor2*, 4- Δ*pta* Δ*aor2* Δ*aor1*, 5- Δ*pta* Δ*adhE1* Δ*adhE2*. The lower bands in the lanes correspond to expected gene deletions targeted by the homology arms.

If *pta* is not essential for acetate formation, how is acetate enzymatically produced in the Δ*pta* strain? There are a number of enzymes identified that can influence acetyl-CoA that may change product formation, in particular AOR and the AdhE. We therefore targeted these genes using CRISPR/Cas9 to understand their metabolic function (Figure 3). We show deletion of these genes using PCR to amplify external target gene regions (Figure 3C) and confirmed loss of the genes by PCR and sequencing (Supplementary Figures S1, S2).

AOR catalyzes the fully reversible interconversion of acetate and acetaldehyde with ferredoxin as the electron donor or acceptor. Although reversible, AOR’s physiological directionality is of question but could be the source of acetate, if acetaldehyde is derived from acetyl-CoA (Ueki et al., 2014; Richter et al., 2016; Nissen and Basen, 2019). Transcriptomics studies under heterotrophic and autotrophic conditions have shown that *aor2* (CLJU_c20210) is the predominantly expressed AOR (2085 heterotrophic/612 autotrophic FPKM), vs. *aor1* (CLJU_c20110) (10 heterotrophic/7 autotrophic FPKM) (Nagarajan et al., 2013). CRISPRi targeting *aor2* showed an effect on fermentation products, although only while targeting *pta* as well (Woolston et al., 2018). Thus, we decided to first target *aor2* using CRISPR/Cas9 in the Δ*pta* strain background.

**FIGURE 4:**
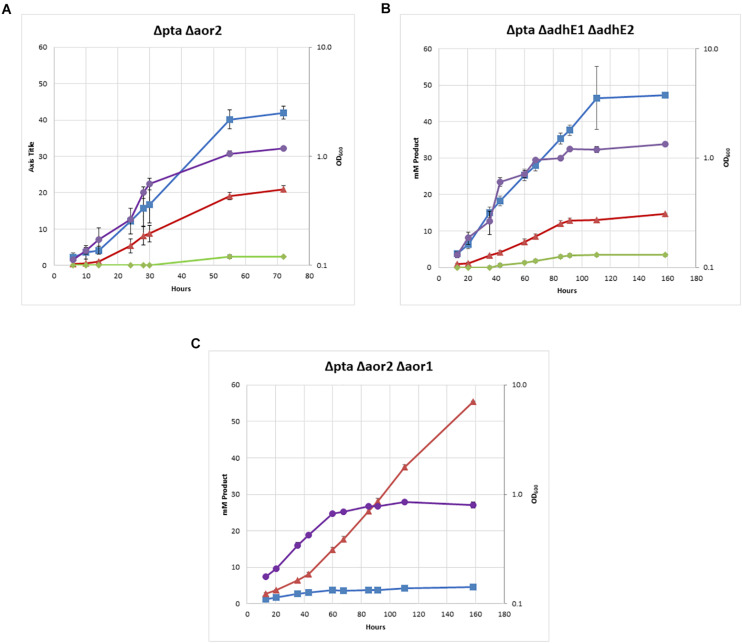
Heterotrophic growth and fermentation products of strains on PETC with 5 g/L fructose. Acetate in blue squares, ethanol in red triangles, 2,3-butanediol in green diamonds, OD_600_ in purple circles. **(A)** Δ*pta* Δ*aor2*, **(B)** Δ*pta* Δ*adhE1* Δ*adhE2*, and **(C)** Δ*pta* Δ*aor2* Δ*aor1.*

The double Δ*pta* Δ*aor2* strain under heterotrophic conditions produced much more ethanol than the wild type or parent Δ*pta* strains. Whereas ethanol was produced in trace amounts in both those strains with an acetate to ethanol ratios of 9:1 (Figure 2), the Δ*pta* Δ*aor2* strain consistently produced acetate to ethanol in a 2:1 ratio (Figure 4A). This suggests that *aor2* directionality under heterotrophic growth at pH 6.0 is in the acetate forming direction. It also suggests *aor2* was important for reduced ferredoxin production and/or acetaldehyde oxidation and prevented ethanol formation under heterotrophic conditions. In contrast to the Δ*pta* strain, the Δ*pta* Δ*aor2* strain under these conditions produced similar 2,3 BDO (2 mM) to the wild type, despite an increase in the production of ethanol. The carbon/electron recovery from fermentation products was 0.82/0.97 for the Δ*pta* Δ*aor2* strain.

Next, we decided to target the bifunctional alcohol/aldehyde dehydrogenases (*adhE*) genes in the Δ*pta* strain. Bifunctional alcohol/aldehyde dehydrogenases catalyze the conversion of acetyl-CoA to acetaldehyde to ethanol using 2 NAD(P)H as electron donors, and could be the source of acetaldehyde substrate for AOR. These genes are also considered important for ethanol production in other *Clostridia*, so the production of both acetate and ethanol could be affected (Cooksley et al., 2012; Lo et al., 2015). There are two annotated *adhE* genes in the genome, *adhE1* and *adhE2* (CLJU_c16510 and CLJU_c16520), which are colocalized in the genome. It was previously shown that disruption of *adhE1* but not *adhE2* resulted in a decrease in ethanol when grown on fructose (Leang et al., 2013). Omics studies have also shown that this is probably because *adhE1* is the predominantly expressed AdhE (166 heterotrophic/0.1 autotrophic FPKM) vs. *adhE2* (5.3 heterotrophic/0 autotrophic FPKM) (Nagarajan et al., 2013). Due to colocalization of *adhE1* and *adhE2*, we decided to simultaneously target both to create the strain Δ*pta* Δ*adhE1* Δ*adhE2* (Figure 3C). We targeted *adhE1* using the guide RNA and supplied recombination regions downstream of the *adhE2* for repair. The Δ*pta* Δ*adhE1* Δ*adhE2* strain showed increased ethanol formation (15 mM) over the parent Δ*pta* strain (∼5 mM), suggesting that there are additional proteins involved in ethanol formation, perhaps from other annotated acetaldehyde/alcohol dehydrogenases (Figure 4B; Whitham et al., 2015). The acetate amount is comparable to that of the Δ*pta* parental strain. The carbon/electron recovery from fermentation products was 0.83/0.94 for the Δ*pta* Δ*adhE1* Δ*adhE2* strain.

Finally, we targeted *aor1* in the Δ*pta* Δ*aor2* strain. Whether *aor1* had an influence on metabolism in *C. ljungdahlii* was unclear in literature. Low detection of *aor1* was shown in a previous study (10.4 heterotrophic/6.5 autotrophic FPKM) (Nagarajan et al., 2013), although there were reports of increased *aor1* under some conditions and variability in some reports (Whitham et al., 2015; Richter et al., 2016; Aklujkar et al., 2017). To target *aor1* we decided to test the original Cas9 construct vs. a riboswitch inducible Cas9 recently developed (Marcano-Velazquez et al., 2019). Working with Cas9 construct for the previous deletions was difficult due to several reasons. In *E. coli*, the plasmid was unstable and often produced truncated plasmids, and did not readily produce concentrated plasmid. In *C. ljungdahlii* transformation efficiencies were low due to constitutive expression of Cas9, which would only allow growth of colonies that had both taken up the plasmid and undergone successful genome editing (Huang et al., 2016). Coupling of an inducible riboswitch with Cas9 has shown improved genomic editing in other *Clostridia* (Cañadas et al., 2019). Switching the original thiolase promoter with the riboswitch inducible promoter (the 2-AP riboswitch linked with the Cthe_2638 promoter, pbuE, *P* = 8 bp) resulted in improved performance in *E. coli* and allowed comparable transformation efficiency of non-Cas9 plasmids, while we were unable to get successful transformants of the constitutive Cas9 construct targeting *aor1*. After getting successful colonies, we induced Cas9 using 2-AP and plated cells on 2-AP plates. Five colonies were isolated and tested, with two showing the edited *aor1* genotype.

The Δ*pta* Δ*aor2* Δ*aor1* strain showed several distinct features compared to Δ*pta* Δ*aor2* (Figure 4C). There was a dramatic decrease in acetate and increase in ethanol, with a ∼85% selectivity for ethanol compared to acetate. No 2,3 BDO was detected. The strain also grew much slower than other strains, including Δ*pta* Δ*aor2*, but was eventually able to consume all the fructose. The carbon/electron recovery from fermentation products was 0.72/1.05 for the Δ*pta* Δ*aor2* Δ*aor1* strain. To confirm the acetate phenotype, the Δ*pta* Δ*aor2* Δ*aor1* strain was retransformed with the control plasmid or a plasmid expressing *pta*/*ack* or *aor2*, which resulted in increased acetate vs. Δ*pta* Δ*aor2* Δ*aor1* with the control plasmid (Supplementary Figure S3). The plasmid complementation did not completely restore acetate production, but that may be due to inferior expression from plasmids (Leang et al., 2013).

### Enzyme Assays on Strains

To examine changes brought by targeted gene deletions, we assayed strains for enzymatic activity based on the targeted genes. First, we measured phosphotransacetylase activity of the wild type and Δ*pta* strain and confirmed loss of detectable phosphotransacetylase activity, measuring wild type specific activity of 1.47 vs. < 0.01 for the Δ*pta* strain. Next, we measured the activity of AOR, Aldh, and Adh (Table 1). For AOR activity, we measured relatively low activity in the wild type strain, which increased fivefold in the Δ*pta* strain. Interestingly, the Δ*pta* Δ*aor2* strain had the highest detectable AOR activity, while the Δ*pta* Δ*aor2* Δ*aor1* strain had the lowest activity of the Δ*pta* derivative strains, but still significantly higher activity than the wild type. For Aldh NAD^+^ activity, we found that a large increase in the Δ*pta* strain vs. the wild type, and a subsequent reduction in Aldh activity in the Δ*pta* Δ*adhE1* Δ*adhE2*, although still above wild type levels. For ADH activity, activities were low for wild type, Δ*pta*, and Δ*pta* Δ*adhE1* Δ*adhE2*, although the highest activity was found in the Δ*pta* Δ*adhE1* Δ*adhE2*.

**TABLE 1:** AOR, Adh, and Aldh cell free extract specific activity.

	**BV AOR**	**NAD^+^ ADH**	**NADP^+^ ADH**	**NAD^+^ ALDH**	**NADP^+^ ALDH**
WT	0.09 (0.00)	0.01 (0.01)	0.03 (0.01)	0.01 (0.01)	0.01 (0.01)
pta	0.38 (0.02)	0.01 (0.01)	0.00 (0.00)	0.46 (0.01)	0.01 (0.01)
pta aor2	0.47 (0.02)	ND	ND	ND	ND
pta aor2 aor1	0.31 (0.02)	ND	ND	ND	ND
pta adhE1 adhE2	ND	0.03 (0.01)	0.01 (0.01)	0.08 (0.01)	0.01 (0.01)

**Cell free extract activities of acetaldehyde ferredoxin oxidoreductase, alcohol dehydrogenase, and aldehyde dehydrogenase, reported in μmol min^–1^ mg^–1^, measured as reduction of the electron acceptor. The top line lists the electron acceptor, either NAD(P)^+^ or benzyl viologen (BV). AOR, aldehyde ferredoxin oxidoreductase; ADH, alcohol dehydrogenase; ALDH, aldehyde dehydrogenase; ND, not determined. Standard deviation is in parenthesis. For all assays, n = 3.**

### Autotrophic Growth of Strains

**FIGURE 5:**
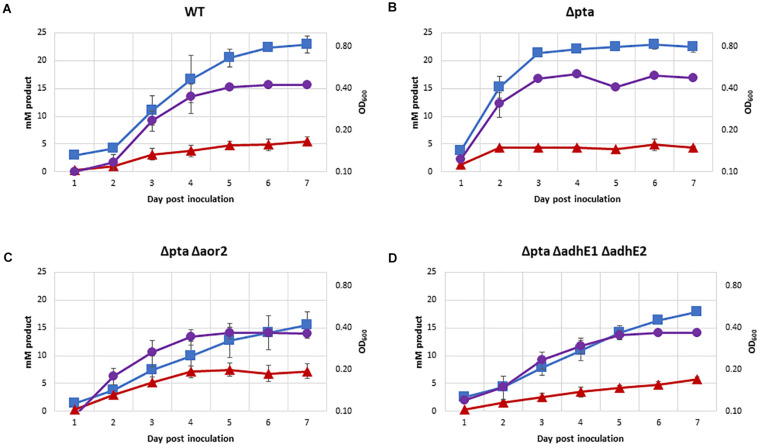
Growth and fermentation products of strains on PETC with 100% CO. Strains were grown over 7 days with fermentation products and growth monitored, with approximately a 5:1 headspace to liquid ratio. Acetate as blue squares, ethanol as red triangles, OD_600_ as purple circles. **(A)** Wild type, **(B)** Δ*pta*, **(C)** Δ*pta* Δ*aor2*, and **(D)** Δ*pta* Δ*adhE1* Δ*adhE2.*

One of the most important predicted functions of Pta in *C. ljungdahlii* is to generate acetyl-P for ATP synthesis. The previous CRISPR/Cas9 study on the Δ*pta* strain showed no significant growth on syngas (Huang et al., 2016). In contrast with previously data, we demonstrated robust growth of most strains on 100% CO in PETC media without sugar over a 7-day period (Figure 5), except for Δ*pta* Δ*aor2* Δ*aor1* which did not grow (OD did not increase by more than 0.1 over 7 days). Acetate was the primary product formed in all strains, with Δ*pta* Δ*aor2* having the least (16 mM) and wild type and Δ*pta* the most (∼23 mM). Δ*pta* Δ*aor2* had the most (7 mM) of ethanol formed, while the others had similar levels (∼5 mM). 2,3 BDO was not a significant product (<2 mM formed). Final OD of strains ranged between 0.35 and 0.48, with strains that generated more acetate having a higher OD.

#### H_2_-Enhanced Mixotrophic Growth for Carbon Conversion

Traditional fermentation ethanol yields are limited to a theoretical 66% carbon yield due to loss of CO_2_ via decarboxylation when converting pyruvate to acetyl-CoA. Acetogens can overcome these limitations by simultaneously utilizing fructose and syngas in mixotrophic growth, allowing for capture of CO_2_ and conversion to acetyl-CoA (Jones et al., 2016). *C. ljungdahlii* was shown to be one of the best acetogens at syngas-enhanced mixotrophic growth as well as ethanol tolerance.

Since the Δ*pta* Δ*aor2* Δ*aor1* was able to produce ethanol at a high yield heterotrophically, we were interested in its performance under H_2_-enhanced mixotrophic growth compared to the wild type. Theoretically H_2_ could be used to capture lost CO_2_ from glycolysis and supply reducing power to reduce acetyl-CoA to ethanol, resulting in a carbon yield increase on a per sugar basis. In a wild type strain, addition of H_2_ with growth on sugar was shown to shift fermentation products toward more ethanol (Jones et al., 2016).

**FIGURE 6:**
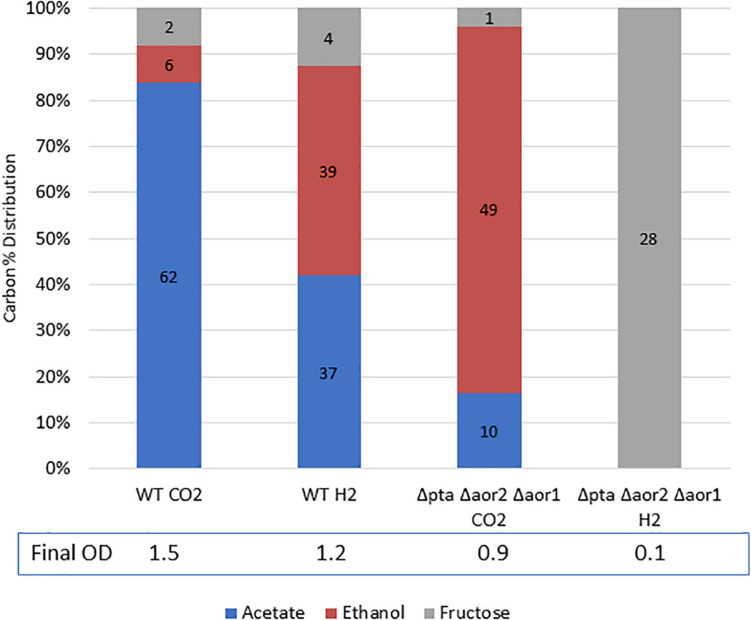
Mixotrophic growth and fermentation products of wild type vs. Δ*pta* Δ*aor2* Δ*aor1* on PETC with 5 g/L fructose with either 100% CO_2_ or 100% H_2_. Strains were grown on 5 g/L (28 mM) fructose with the indicated headspace gas (CO_2_ or H_2_). Carbon distribution of fermentation products/residual fructose is indicated by bar chart%, while the number label is mM amount after 5 days. The OD_600_ after 5 days is indicated at the bottom.

While the Δ*pta* Δ*aor2* Δ*aor1* grew heterotrophically but not on CO alone, we wondered whether the strain could utilize fructose and H_2_ simultaneously to get a larger ethanol yield than on fructose alone. To test this, we grew the wild type strain and the Δ*pta* Δ*aor2* Δ*aor1* strain on 5 g/L fructose with either 100% H_2_ or 100% CO_2_ (as a control) in the headspace (Figure 6). After a week of growth, the wild type with CO_2_ or H_2_ and Δ*pta* Δ*aor2* Δ*aor1* with CO_2_ consumed most of the sugar, while the Δ*pta* Δ*aor2* Δ*aor1* with H_2_ failed to grow at all. Based on the fructose added, the carbon recovery of the wild type strain was ∼90% in CO_2_ and ∼105% in H_2_, while only 75% Δ*pta* Δ*aor2* Δ*aor1* in CO_2_.

## Discussion

Acetate formation is a key characteristic of acetogens, which utilize the WLP to fix CO_2_ and produce primarily acetate. Surprisingly under a wide variety of conditions in *C. ljungdahlii*, Pta is unnecessary for acetate formation, despite being normally understood as a critical enzyme for ATP production and acetate formation. What are the underlying mechanisms and enzymes that drive *C. ljungdahlii* metabolism? There are a few models that attempt to predict *C. ljungdahlii* metabolism. Nagarajan’s genome-scale model suggested that deleting *pta* and *ack* would force ethanol production rather than acetate, but this model was missing the AOR reaction functioning in the acetate forming direction (Nagarajan et al., 2013). Richter et al. (2016) suggested that thermodynamics, rather than enzyme expression, is a driving force behind the product formation in *C. ljungdahlii*, and that AOR is an important arbiter in determining acetate:ethanol ratios. We provide evidence that model has value. Despite *pta* as one of the most highly expressed genes and an important contributor to ATP/acetate formation, the Δ*pta* and wild-type strain had very similar product profiles. Richter et al. (2016), however, suggested that a *pta/ack* knockout strain would not produce acetate, but we have shown high acetate yield without *pta*. We believe this is through the activity of AOR. AOR has been proposed to be important for metabolism in *C. ljungdahlii*, and *aor2* has been found to be expressed under both heterotrophic and autotrophic growth conditions (Nagarajan et al., 2013; Ueki et al., 2014; Whitham et al., 2015; Richter et al., 2016; Aklujkar et al., 2017; Woolston et al., 2018; Zhu et al., 2020). What is the purpose of AOR in acetogens? There are several potential functions: ferredoxin reoxidation under growth on CO, detoxifying short-chain fatty acids when pH is low, and maximizing ATP yield during autotrophic ethanol production (Mock et al., 2015; Richter et al., 2016; Liew et al., 2017).

We provide evidence that a potential function of AOR may be to supply ferredoxin via an acetaldehyde intermediate from acetyl-CoA. Although in acetogens, AOR was thought to primarily function in the reduction of carboxylic acids, oxidation of aldehydes may be an important feature as well. Balancing redox cofactors is likely to be an important part of *C. ljungdahlii* metabolism and AOR could be a method of indirectly “upgrading” electrons from NAD(P)H (E_0_’ = −320 mV) to Fd_red_ (E_0_’ = −400 mV). The directionality of AOR is partially determined by pH, as AOR is only active on protonated short-chain fatty acids (i.e., acetic acid, pKa = 4.7) (Huber et al., 1995; Napora-Wijata et al., 2014). A higher pH would drive the reaction toward reducing ferredoxin as acetic acid would be deprotonated to acetate. High expression of AORs has been shown in both heterotrophic and autotrophic conditions, despite high yields of acetate, so even in Pta and Ack competent strains AOR may be converting acetaldehyde to acetate. This process would provide redox flexibility and ATP through the conversion of Fd_red_ to NAD(P)H via the NADH ferredoxin:NADP^+^ oxidoreductase and proton-translocating ferredoxin:NAD^+^ oxidoreductase, which would then be consumed by the electron demanding WLP. Indeed, in many of our Δ*pta* strains, growth and product formation was like the wild type and only appeared to be severely compromised when both *aor1* and *aor2* were also deleted (Figure 4C). Interestingly, enzyme assays in the Δ*pta* strain of both NAD^+^-linked Aldh and AOR were significantly higher than the wild type strain, suggesting that this pathway is the main route of acetate formation in this strain. Even with loss of *aor2*, we detected the highest AOR activity in the Δ*pta* Δ*aor2* strain (Table 1). It should be noted that the AOR assay used benzyl viologen rather than ferredoxin, and thus these activities may not be true indicators of AOR activity. AOR genes are widely distributed among acetogens and could serve a similar role in those organisms (Nissen and Basen, 2019). In the closely related *Clostridium autoethanogenum*, *aor1*, and *aor2* were deleted, and it was shown that loss of *aor2*, but not *aor1*, improves autotrophic ethanol production. In the same study, it was also shown that loss of these genes prevents reduction of carboxylic acids (Liew et al., 2017). It is unknown why there is a difference in the response between *C. ljungdahlii* and *C. autoethanogenum*, but potentially it was because the *pta/ack* pathway was functional in those strains. Pta and Ack are usually some of the highest detected enzymes, which points to their central role in acetate formation. In our Δ*pta* derivative strains, AOR may be the only functional route to acetate, which may be a preferred product due to electron consumption by the WLP. It is still unknown why there are two genes and what factors drive their expression. Additionally, while these organisms are over 99% identical, there are significant differences in metabolism, substrate consumption, and product formation (Bruno-Barcena et al., 2013; Jones et al., 2016). Specific differences in the WLP enzymes include a uniquely truncated *C. autoethanogenum acsA* gene which could have a major effect on metabolism (Liew et al., 2016).

The Δ*pta* Δ*aor2* Δ*aor1* strain shows slower heterotrophic growth. Why is this the case? The WLP is an electron demanding process and the formation of ethanol would potentially cause redox imbalance. Alternatively, if AOR “upgrading” of electrons is disabled, there may be a cofactor mismatch between electrons produced by glycolysis (Fd_red_ and NADH) and demanded by the WLP. Electron requiring enzymes of the WLP require redox cofactors (i.e., NADPH, Fd_red_, and possibly NADH) in specific amounts. Fd_red_ can be used to reduce NAD(P)^+^ through the activity of Rnf and Nfn, which has been detected in high activities, suggesting these interconversions are actively taking place.

The Δ*pta* Δ*aor2* Δ*aor1* strain also showed no detectable autotrophic growth, even though ethanol formation through *adhE* is a net ATP positive process (Bertsch and Müller, 2015; Mock et al., 2015). We suspect that this is due to the regulation of *adhE1*, as *adhE1* appears to be highly expressed under heterotrophic conditions but virtually undetectable in autotrophic conditions (Nagarajan et al., 2013). This makes sense as ethanol production through ADHE is much less ATP efficient than through AOR, but the underlying regulatory mechanism of *adhE* control is still unknown. Mixotrophic conditions of Δ*pta* Δ*aor2* Δ*aor1* also showed no detectable growth. We think this may be due to CO/H_2_ inhibiting strong expression of *adhE1*, like on autotrophic growth. If this is the case, *adhE1* regulation may not be an autotrophic mechanism *per se*, but rather in response to reducing gases or redox state, regardless of the presence of sugar. Alternatively, a recent study looking at *C. autoethanogenum* autotrophic growth suggests that ethanol formation through ADHE may be thermodynamically unfeasible under certain conditions and that AOR is important for energy generation and redox balance (Mahamkali et al., 2020). These factors could explain the lack of growth in the presence of syngas. Although there have been studies looking at heterotrophic, autotrophic, and mixotrophic growth, little is known about the mechanisms that govern the metabolism of these organisms. Since acetogenic metabolism is ATP and thermodynamically limited, there may be multiple mechanisms at work (Schuchmann and Müller, 2014; Molitor et al., 2017).

The Δ*pta* Δ*adhE1* Δ*adhE2* strain raises several questions around ethanol and acetate formation in this organism. Despite loss of these highly expressed enzymes under heterotrophic conditions, loss of these enzymes increased ethanol formation and significant amounts of ethanol and acetate were formed. Acetate was still the primary product, which is probably from the active AORs in this strain, but ethanol was about 33% of the product formed. AdhE’s main function is probably the reversible conversion of acetyl-CoA to ethanol (and vice versa) and not acetaldehyde generation for AOR activity. AdhEs are known to form spirosomes, which are large structures believed to sequester the toxic acetaldehyde from the rest of the cell (Kim et al., 2019). However, the Δ*pta* strain had higher Aldh activity than the Δ*pta* Δ*adhE1* Δ*adhE2* strain, suggesting that these *adhE* genes may be important for Aldh activity, which has been seen in other *Clostridia* (Yao and Mikkelsen, 2010). Even with the loss of both *adhE* genes, the Δ*pta* Δ*adhE1* Δ*adhE2* strain had some Aldh activity, and slightly higher Adh activity, which could explain the increased ethanol formation. This suggests the presence of other important aldehyde and alcohol dehydrogenases. There are many others annotated and expressed, but their function and importance are not well understood (Whitham et al., 2015). Other studies have pointed out other potential candidates that are highly expressed, but as we have shown for *aor2* vs. *aor1*, expression levels under those studies may be deceptive for predicted metabolic effect. The metabolic effect of these enzymes change depending on the state of cells. For instance, during growth on syngas, ethanol can be both produced and consumed, which is dependent on both AdhE and AOR (Liu et al., 2020). Additionally, there are many potential redundant enzymes and their regulation is unknown, and while many of these genes are annotated as alcohol or aldehyde dehydrogenases, they may not be specific for acetaldehyde/ethanol (Tan et al., 2015). In general, we did not detect high Adh activity, which suggests a lack of highly expressed/active alcohol dehydrogenases. The leftover acetate in the Δ*pta* Δ*aor2* Δ*aor1* fermentations could perhaps be formed from other annotated AORs or from *ctf* (Ueki et al., 2014; Whitham et al., 2015). While we noted strain differences in enzymatic activity, these assays were extremely limited. We did not test cells under different growth conditions or attempt to optimize enzymatic activity, and we used benzyl viologen as a proxy for ferredoxin. A more careful enzymatic study may be expected to find different results (Lo et al., 2015; Mock et al., 2015). It will be important to rigorously test enzymatic activity and identify responsible enzymes, for both metabolic engineering and fundamental understanding.

Understanding the underlying mechanisms of acetogen metabolism is important for engineering them for targeted chemical formation. Acetogens are being investigated for their autotrophic, heterotrophic, and mixotrophic growth capabilities to produce a wide variety of products with high carbon-conversion efficiency. We show that substrate level phosphorylation of ATP via *pta* is not required for robust growth on CO. This may be helpful when designing pathways for product formation, as ATP production via substrate level phosphorylation is not an absolute requirement and ATP production through proton motive force generated by Rnf is sufficient (and necessary) for autotrophic growth (Tremblay et al., 2013). We did not test autotrophic growth differences of the strains on H_2_/CO_2_, which is expected to have lower ATP yield than CO growth, but H_2_/CO_2_ growth is still expected to be ATP positive assuming acetate is formed (Mock et al., 2015).

Indeed, *C. ljungdahlii* has already been engineered to produce acetone, butanol, and butyrate (among many others), and acetate has been an ever-present product in those studies. We have successfully reduced acetate production by > 80% via targeted gene knockout, which should help inform further work in engineering new products in acetogens and increasing yield of desired products. In this work, we engineered *C. ljungdahlii* and improved yield of ethanol from ∼10% to over 80%. In the process, we clarified the role of several genes involved in acetyl-CoA metabolism and showed that substrate phosphorylation via *pta* is unnecessary for autotrophic growth on CO (Figure 5). We also identify gaps in our knowledge of acetogens and highlight important areas of study in acetogens for further research in both fundamental understanding and applied metabolic engineering.

## Author’s Note

This work was authored by the National Renewable Energy Laboratory, operated by Alliance for Sustainable Energy, LLC, for the U.S. Department of Energy (DOE) under Contract No. DE-AC36-08GO28308. Funding provided by DOE and Bioenergy Technology Office (BETO). The views expressed in the article do not necessarily represent the views of the DOE or the U.S. Government. The U.S. Government retains and the publisher, by accepting the article for publication, acknowledges that the U.S. Government retains a nonexclusive, paid-up, irrevocable, worldwide license to publish or reproduce the published form of this work, or allow others to do so, for U.S. Government purposes.

## Data Availability Statement

All datasets generated for this study are included in the article/Supplementary Material.

## Author Contributions

JL, P-CM, YG, and ZR led the research. JL and JH designed the experiments. JL, JH, JJ, CU, LM, and WX performed the experiments. JL, WX, and P-CM wrote the article. All authors contributed to the article and approved the submitted version.

## Conflict of Interest

The authors declare that the research was conducted in the absence of any commercial or financial relationships that could be construed as a potential conflict of interest.
