# Supplementary material for: The Metabolism of Clostridium ljungdahlii in Phosphotransacetylase Negative Strains and Development of an Ethanologenic Strain
Source: Front Bioeng Biotechnol. 2020 Oct 27;8:560726. doi: 10.3389/fbioe.2020.560726 (PMC7653027; doi:10.3389/fbioe.2020.560726)
Supplement: Supplementary file 1 [file Data_Sheet_1.docx]

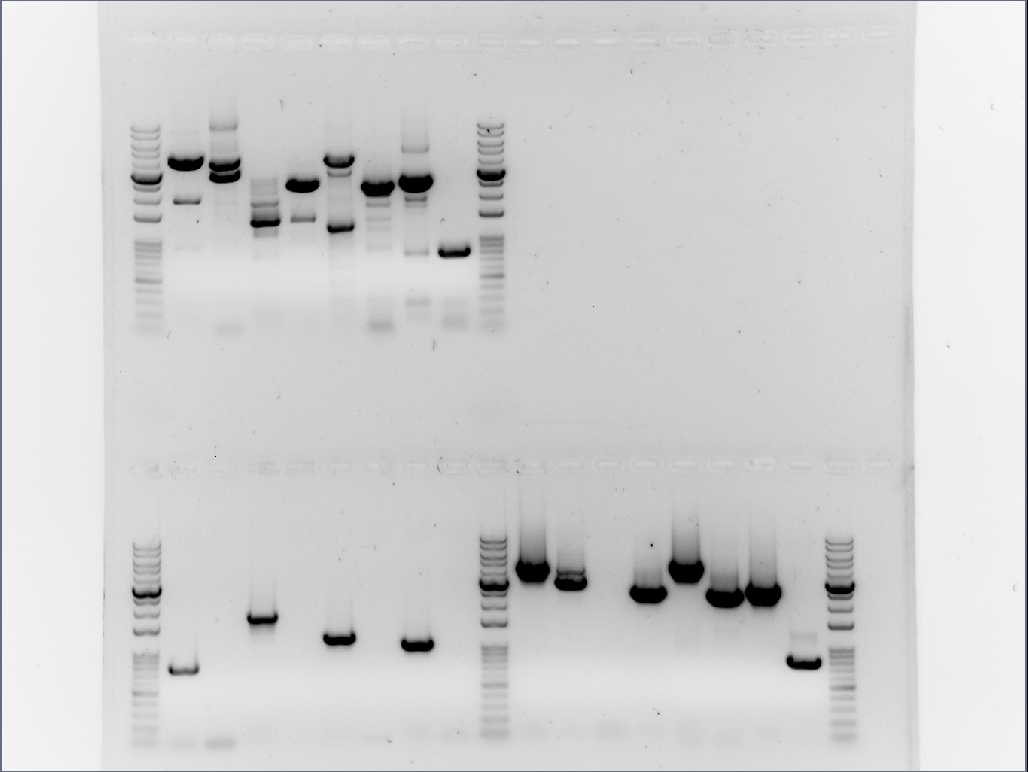


*pta*

*aor2*

*aor1*

*adhE1/2*

L wt 2 wt 3 wt 4 wt 5 L

Figure S1. PCR amplifying internal fragments from Wild type(wt) and mutant strains, showing the presence of a gene in the wt, but loss in the deletion strain. L is the ladder. 2 is ∆*pta*, 3 is ∆*pta* ∆*adhE1* ∆*adhE2*, 4 is ∆*pta* ∆*aor2,* 5 is ∆*pta* ∆*aor2* ∆*aor1*. Primer sequences are in Table S1.


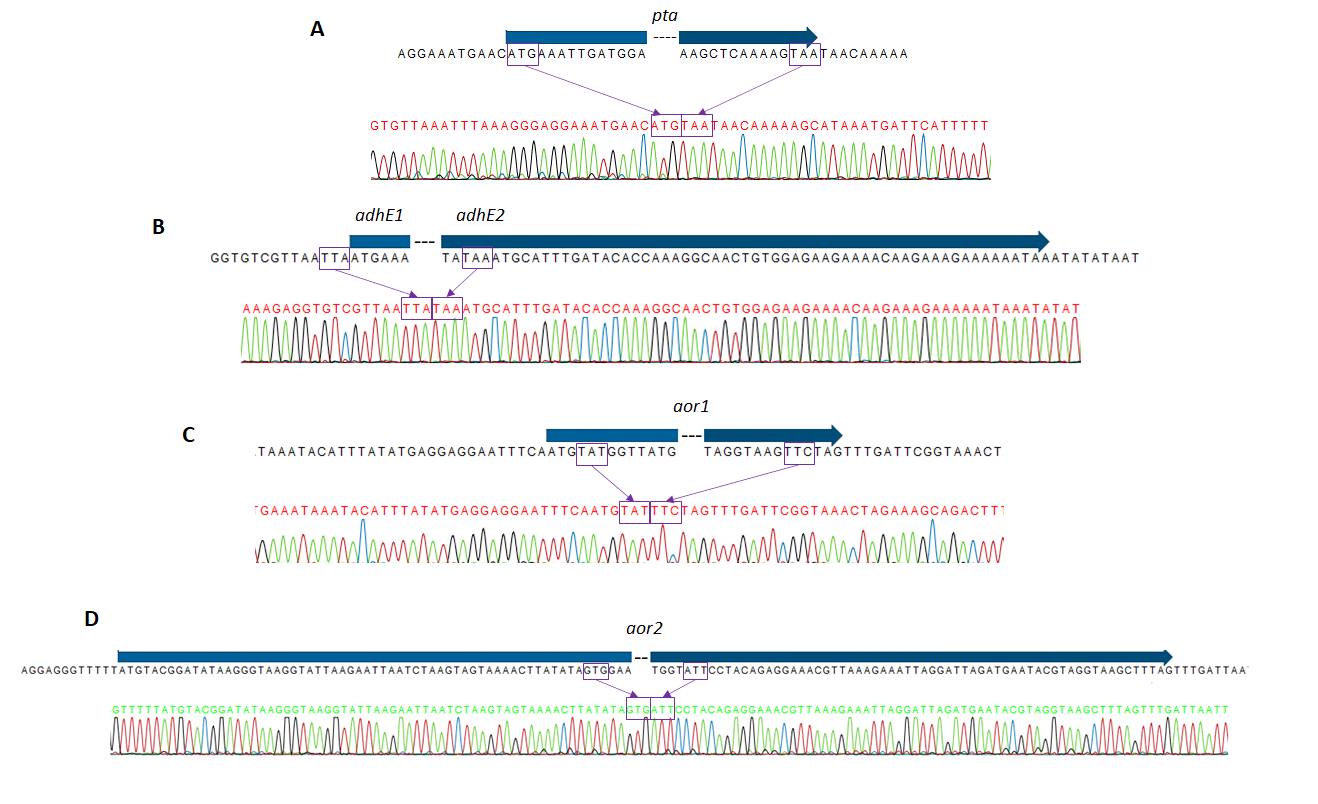


Figure S2. Sanger sequencing results of genomic regions showing deletions. Gene names are above blue arrows. Boxes outline where the deletions start and end in relation to the gene. The blue arrows indicate the location of the genes. A. *pta* deletion region. B. *adhE1* and *adhE2* deletion region. C. *aor1* deletion region. D. *aor2* deletion region.

Figure S3. Complementation of ∆*pta* ∆*aor2* ∆*aor1*. Strains were grown on 5 g/L fructose for 144 hours and were measured for their fermentation products. pMTL83151 is the control plasmid. pta/ack is the plasmid expressing *pta* and *ack* under the native promoter. aor2 is the plasmid expressing *aor2* under the native promoter. Both complementation strains show increased acetate, with *pta/ack* complementation showing double the acetate of ∆*pta* ∆*aor2* ∆*aor1*, while *aor2* complementation showed 50% more acetate. Error bars are standard deviation, n=3.
